# Whole cottonseed inclusion in starter feeds improves performance, inflammometabolic profile, and rumination behavior in Holstein dairy calves

**DOI:** 10.3168/jdsc.2022-0368

**Published:** 2023-07-21

**Authors:** Annalisa Amato, Andrea Minuti, Luigi Liotta, Luca Cattaneo, Marta Sfulcini, Erminio Trevisi, Vincenzo Lopreiato

**Affiliations:** a Department of Veterinary Sciences, Università di Messina, 98168 Messina, Italy; b Department of Animal Science, Food and Nutrition (DIANA), Faculty of Agricultural, Food and Environmental Sciences, Università Cattolica del Sacro Cuore, 29122 Piacenza, Italy; c Romeo and Enrica Invernizzi Research Center for Sustainable Dairy Production of the Università Cattolica del Sacro Cuore (CREI), 29122 Piacenza, Italy

## Abstract

The high energy, protein, and fiber contents of whole cottonseed make it a potential candidate for the inclusion in calf starters to promote the rumen development. This study aimed at assessing whether the inclusion of whole cottonseed in the starter would affect performance, metabolic profile, and rumination time in Holstein dairy calves. From 2 to 55 d of age, 12 heifer calves were fed a constant amount of milk replacer twice daily (8 L/d), whereas from 56 to 65 d (weaning) milk replacer was gradually reduced (from 4 to 1 L/d) and fed in a single meal. Calves were blocked by birth body weight and % Brix of colostrum received and randomly assigned to 1 out of 2 dietary treatments: (1) control starter (CTR); (2) starter with 8% inclusion of whole cottonseed (WCS). Treatments were fed for ad libitum intake. From d 56, TMR and hay were offered ad libitum. At 0, 2, 7, 21, 65, and 80 d, BW was measured and blood samples were collected. Feed intake and rumination time were automatically recorded. Compared with CTR, WCS calves were heavier at weaning and after weaning, and consumed more starter from 59 to 72 d. In the immediate postweaning (from 66 to 72 d of age), rumination time increased more in WCS calves. Overall, WCS calves had greater plasma glucose, β-carotene, and retinol concentrations, whereas ceruloplasmin and myeloperoxidase were lower. Calves in the WCS group had greater glucose concentration at 21 and 65 d and lower urea at 65 d (weaning). Plasma β-carotene concentration was greater at 65 and 80 d in calves of the WCS group compared with CTR calves. At 80 d, WCS calves had lower plasma alkaline phosphatase and greater β-hydroxybutyrate, paraoxonase, and tocopherol. These results suggest that inclusion of WCS in the calf starter might be beneficial for rumen development, leading to greater feed intake and BW. Moreover, WCS inclusion was associated with lower oxidative stress and inflammation, improved energy metabolism and liver functionality, and likely quicker rumen development, as might be indicated by the higher plasma β-hydroxybutyrate and rumination time. These changes occurred mainly after weaning, when no differences in starter intake and average daily gain were detected, suggesting a better efficiency of nutrient utilization at this age.

## Highlights

• Starter at 8% of WCS improved intake, postweaning BW, and feed efficiency of calves.
• WCS improved energy metabolism and liver functionality.
• Feeding WCS resulted in greater plasma β-hydroxybutyrate and longer rumination time.
• Feeding WCS resulted in lower oxidative stress and inflammatory response.

## Graphical Abstract

**Figure:**
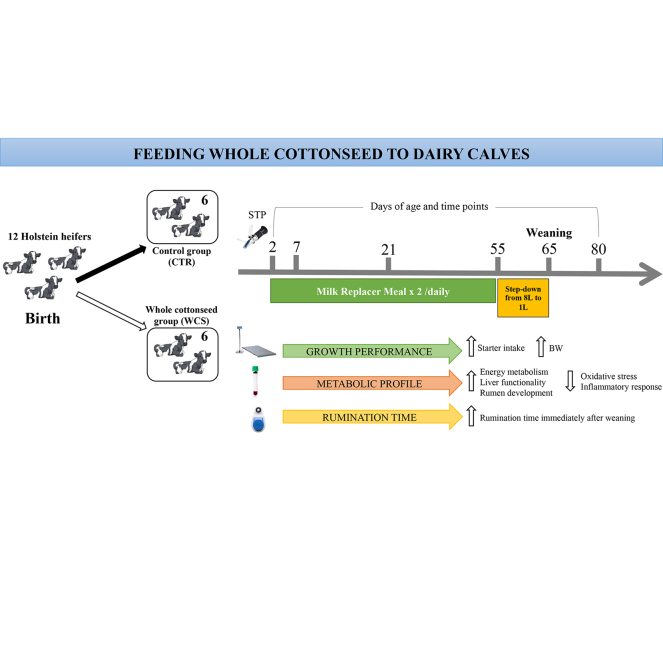
**Summary:** For its fat and protein contents, whole cottonseed (WCS) may be defined as a concentrate. On the other hand, its fiber is similar to that of forages in terms of effectiveness in the rumen. Considering the high digestibility of WCS fiber, its inclusion in calf starter could improve growth and rumen development. Hence, we investigated the effect of including WCS at 8% on growth performance, inflammometabolic profile, and rumen behavior in Holstein heifer calves. Calves receiving WCS in the starter showed increased average body weight (BW), improved metabolic profile, and longer rumination time compared with the control group. These results suggest that WCS may be included as a starter ingredient with beneficial effects.

There is a growing interest for new ingredients to be included in the calf diet to prepare animals for weaning. Starters should be high in readily fermentable carbohydrates and adequate in digestible fiber to support rumen development at a time when cellulose digestibility is limited (NRC, 2001). In this context, cottonseed is considered an excellent addition to ruminant feed (Anderson et al., 1982; Lima et al., 2017). Cottonseed is an oilseed, rich in UFA such as linoleic acid, but also rich in protein with a good AA profile and cellulose with high digestibility. Cottonseed is used mainly in lactating cows but its application in growing animals is scarce. In a previous study, Anderson et al. (1982) reported that feeding whole cottonseed in young calves resulted in greater feed intake, BW, and rumen papillae development. Indeed, its fiber is similar to that of forages in terms of effectiveness in the rumen (Anderson et al., 1982). Therefore, because of its chemical composition (NRC, 2001), cottonseed can represent a potential candidate for the inclusion in the calf diet. We hypothesized that whole cottonseed inclusion in calf starter could improve growth and rumen development because of its high digestibility of fiber content. Hence, the aim of this study was to evaluate the effects of whole cottonseed inclusion on performance, metabolic profile, feeding behavior, and rumination time.

All procedures were approved by the Università Cattolica Animal Welfare Committee and carried out in accordance with Italian laws on animal experimentation (DL n. 26, 04/03/2014; Ministerial authorization no. 123/2022 PR). The study was performed in a commercial dairy farm in Northern Italy. Twelve female Holstein dairy calves were enrolled over a period of 15 d, within 1 h from birth, after being separated from their dams and moved to individual outdoor hutches bedded with straw. Calves were cleaned, weighed, had the navel disinfected with oxytetracycline hydrochloride (Neo Spray Caf Aerosol; Gellini S.p.a., Aprilia, Italy). All calves received 2 L of colostrum soon after birth and another 2 L, as second colostrum meal, 12 h after birth. Colostrum used in the experiment was collected and frozen in the months preceding the trial and % Brix of colostrum was measured with a handheld refractometer (PU-ATC temperature compensated; Kernco Instruments Co.). Only colostrum with 25% Brix and above was stored. The experiment was performed as a randomized controlled trial. Calves were blocked by birth BW and Brix percentage of colostrum administered and then randomly assigned to 1 out of 2 treatments: (1) no inclusion of whole cottonseed in the starter (**CTR**, n = 6) and (2) 8% whole cottonseed inclusion in the starter (**WCS**, n = 6). Starter fed CTR calves had 87.8% DM, 20.5% CP, 4.6% ether extract, and 24.6% NDF, whereas WCS starter had 87.8% DM, 18.8% CP, 6.3% ether extract, and 27.3% NDF. Calves received whey-based milk replacer (**MR**; 23.1% CP and 19% fat; Denkavit Italia S.R.L., Italy) twice daily (0800 and 1600 h) at a rate of 125 g/L of water until 55 d of age: 4 L/d from 2 to 7 d, 5 L/d from 8 to 15 d, 6 L/d from 16 to 20 d, and 8 L/d from 21 to 55 d. The step-down weaning started at 56 d and calves were completely weaned at 65 d. Fed and refused MR was recorded at each meal. Each calf was fitted at 2 d of age with a 3-axis accelerometer ear-tag (Allflex Livestock Intelligence, Merck & Co. Inc., Rahway, NJ, and its affiliates) to quantify daily rumination time. Starters were offered for ad libitum intake from 4 d of age to weaning once every morning after MR feeding. Moreover, from 56 d of age (i.e., beginning of the weaning period), grass hay and TMR (made of 16% DM of grass hay, 51% DM of grass haylage, and 33% DM of CTR starter) were also offered ad libitum. Newly fed and refused starter, hay, and TMR were recorded daily.

Blood samples were collected from the jugular vein into heparinized tubes before the morning MR meal on 2, 7, 21, 65, and 80 d of age. Tubes were immediately cooled in an ice-water bath and then centrifuged at 3,500 × *g* for 15 min at 4°C. Plasma was harvested, divided into aliquots, and stored at −20°C. In addition, an aliquot of plasma from blood collected at 2 d of age was used to measure total protein with a handheld refractometer (PU-ATC temperature compensated; Kernco Instruments Co.) to assess transfer of passive immunity. The following plasma biomarkers were measured using analysis methods described in Calamari et al. (2016): Ca, P, Mg, Na, K, Zn, glucose, cholesterol, urea, ceruloplasmin, total protein, albumin, globulin, aspartate aminotransferase, γ-glutamyl transferase, alkaline phosphatase, bilirubin, haptoglobin, nonesterified fatty acids, BHB, creatinine, paraoxonase, myeloperoxidase, total reactive oxygen metabolites, thiol groups, advanced oxidation protein products, ferric-reducing antioxidant power, retinol, tocopherol, and β-carotene.

Calves were weighed at 0, 2, 7, 21, 65, and 80 d of age in the morning before the morning meal. Body weight was used to calculate the ADG. Rumination time was automatically recorded (SenseHub; Allflex Livestock Intelligence, Merck & Co. Inc.). After extraction of raw data by the manufacturer, daily rumination time was calculated from 2 to 80 d of age. Energy balance (% of requirements) was calculated as ME intake/ME requirement × 100 (NRC, 2001).

Data were analyzed with SAS software (version 9.4; SAS Institute Inc.). All data were subjected to ANOVA using mixed models for repeated measures (GLIMMIX Procedure; SAS Institute Inc.). The statistical model included dietary treatment, day of age, and their interaction as fixed effects, whereas calves were included as random effect. Pairwise comparisons were carried out using the LSD test of SAS. Comparisons with *P* ≤ 0.05 were considered significant, whereas when 0.10 ≥ *P* ≥ 0.05 they were discussed in the context of tendencies.

All calves were healthy, sampled, and monitored throughout the study. The Brix percentage of colostrum delivered to calves was not different between groups (30.65 vs. 30.33 ± 3.48% for CTR and WCS, respectively; *P* = 0.53). Plasma total protein measured using a handheld refractometer 48 h after colostrum intake averaged 6.08 ± 0.81 g/dL for CTR and 6.81 ± 0.51 g/dL for WCS calves (*P* = 0.12). Overall, BW did not differ between groups, but WCS had greater BW at 65 d (81.83 vs. 75.25 ± 1.86 kg, respectively; *P* = 0.02; Figure 1) and at 80 d (100.75 vs. 93.33 ± 1.86 kg, respectively; *P* = 0.01; Figure 1). Overall, the starter intake of CTR and WCS calves was 0.89 and 0.99 ± 0.12 kg of DM/d, respectively. In particular, starter intake was greater in WCS calves during weaning (56–65 d of age) and during the postweaning (66–78 d of age) compared with CTR calves (treatment [**Trt**] × day; *P* = 0.01; Figure 1). Overall, the energy balance was lower in WCS calves compared with CTR (125 vs. 131 ± 1.99% of requirements; *P* = 0.04). During the postweaning period, WCS calves had greater rumination time than CTR (66‒72 d; 428.4 ± 19.11 vs. 380.02 ± 19.11 min/d, respectively; *P* = 0.04).

**Figure 1:**
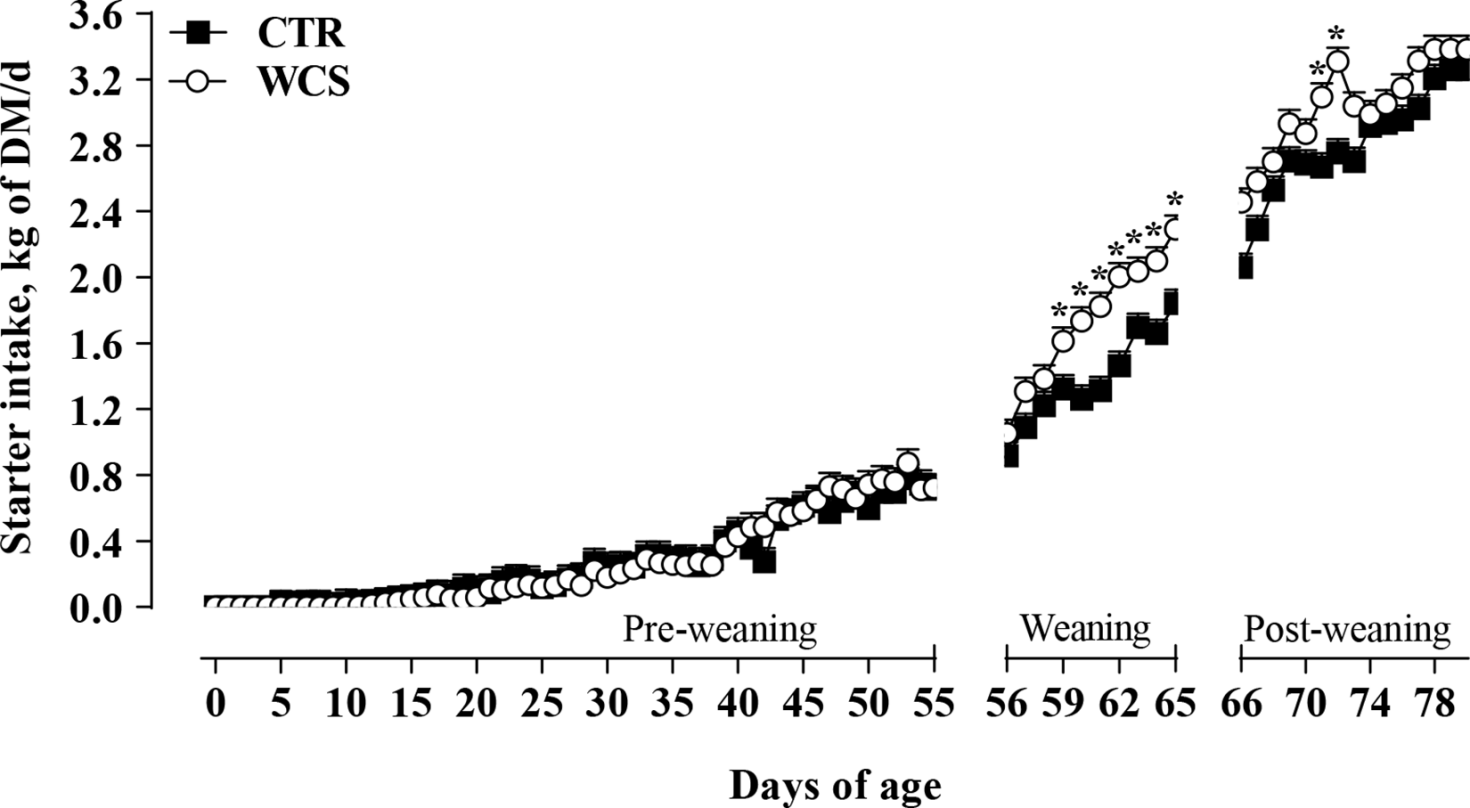
Concentrate intake during preweaning (0–55 d), weaning (56–65 d), and postweaning (66–80 d) of calves fed concentrate without (CTR) or with 8% (WCS) of whole cottonseed inclusion. Error bars indicate the SEM. Asterisks indicate days where concentrate intake differed between treatment groups (*P* < 0.05).

Average plasma biomarker concentrations in the period investigated are reported in Table 1. Glucose concentration (Trt × day, *P* = 0.05) was greater in WCS compared with CTR at 21 (6.53 vs. 5.60 ± 0.27 mmol/L, respectively; *P* = 0.02) and 65 d of age (6.67 vs. 6.03 ± 0.27 mmol/L, respectively; *P* = 0.05; Figure 2). Concentration of BHB (Trt × day, *P* = 0.02) was higher in WCS at 80 d compared with CTR (0.40 vs. 0.29 ± 0.02 mmol/L, respectively; *P* = 0.001; Figure 1), and urea (Trt × day, *P* = 0.07) was lower at 65 d of age (2.19 vs. 4.18 ± 0.46 mmol/L, respectively; *P* = 0.004; Figure 2). Compared with CTR, WCS have lower myeloperoxidase concentration (Trt; *P* = 0.05) and tended to have lower ceruloplasmin (Trt; *P* = 0.10). Among liver function biomarkers, paraoxonase concentration (Trt × day, *P* = 0.10) tended to be greater in WCS at 65 d (86.70 vs. 75.39 ± 5.01 U/mL; *P* = 0.10) and significantly greater at 80 d (94.73 vs. 79.68 ± 5.01 U/mL; *P* = 0.04). Other liver biomarkers were not affected by treatment. Overall, WCS calves tended to have greater retinol (Trt, *P* = 0.08) and significantly greater β-carotene (Trt, *P* = 0.03) concentrations compared with CTR. The interaction Trt × day (*P* = 0.02) affected β-carotene concentrations, resulting at weaning (65 d; 0.02 vs. 0.03 ± 0.003 mg/100 mL, respectively, for CTR and WCS calves; *P* = 0.01) and postweaning (80 d; 0.02 vs. 0.04 ± 0.003 mg/100 mL, respectively, for CTR and WCS calves; *P* = 0.001) higher in WCS than CTR calves. Tocopherol was also affected by the interaction Trt × day (*P* = 0.05), being higher in WCS than CTR calves at 80 d of age (2.27 vs 1.29 ± 0.35 μg/mL, respectively; *P* = 0.05).

**Table 1:** Effects of 8% whole cottonseed inclusion (WCS) into the starter (CTR = control diet) on plasma biomarkers and rumination time of Holstein heifers from 2 to 80 d of age

Biomarker^1^	Diet		SEM^2^	*P*-value^3^		
	CTR	WCS		Trt	Day	Trt × day
Transfer of passive immunity						
Total protein, g/L	68.25	67.76	1.48	0.81	<0.05	0.54
GGT, U/L	295.32	268.79	51.57	0.72	<0.05	0.93
Energy metabolism						
BHB, mmol/L	0.16	0.17	0.01	0.39	<0.05	0.02
NEFA, mmol/L	0.23	0.26	0.02	0.54	<0.05	0.74
Glucose, mmol/L	6.33^y^	6.59^x^	0.09	0.08	<0.05	0.08
Fructosamine, μmol/L	259.77	259.31	4.21	0.94	<0.05	0.13
Creatinine, μmol/L	81.55	85.71	1.91	0.15	<0.05	0.44
Urea, mmol/L	3.72	3.41	0.18	0.26	<0.05	0.07
Liver functionality						
Albumin, g/L	31.34	31.76	0.37	0.45	<0.05	0.69
Paraoxonase, U/mL	45.37	50.58	3.64	0.33	<0.05	0.10
Cholesterol, mmol/L	2.20	2.30	0.16	0.67	<0.05	0.21
Retinol, mg/100 mL	17.75^y^	19.62^x^	0.70	0.08	<0.05	0.71
Alkaline phosphatase, U/L	308.92	289.33	35.43	0.70	0.36	0.01
AST-GOT, U/L	79.27	70.56	3.87	0.14	<0.05	0.95
Bilirubin, μmol/L	7.40	8.56	1.22	0.52	<0.05	0.72
Inflammatory response						
Ceruloplasmin, μmol/L	2.60^x^	2.18^y^	0.16	0.10	<0.05	0.43
Haptoglobin, g/L	0.47	0.42	0.05	0.55	<0.05	0.69
Zn, μmol/L	14.94	14.89	0.89	0.96	<0.05	0.28
Myeloperoxidase, U/L	500.34^a^	452.81^b^	15.61	0.05	<0.05	0.73
Oxidative stress and antioxidant status						
β-Carotene, mg/100 mL	0.02^b^	0.03^a^	0.001	0.03	<0.05	0.02
Tocopherol, μg/mL	2.47	2.47	0.18	0.98	<0.05	0.05
ROM, mg of H_2_O_2_/0.1 L	16.93	14.58	0.98	0.12	<0.05	0.43
Thiol groups, μmol/L	310.44	320.18	13.57	0.62	0.06	0.43
FRAP, μmol/L	137.06	145.27	5.40	0.30	<0.05	0.50
Mineral						
Na, mmol/L	144.84	145.08	0.59	0.78	<0.05	0.59
Ca, mmol/L	2.86	2.88	0.03	0.76	<0.05	0.39
Mg, mmol/L	0.87	0.87	0.01	0.72	<0.05	0.35
K, mmol/L	5.34	5.24	0.09	0.46	<0.05	0.11
P, mmol/L	3.02	2.97	0.07	0.70	<0.05	0.34
Rumination time, min/d	215.20	216.60	20.22	0.95	<0.05	0.05

a,b Different superscripts within a row and specific variable indicate that means differ at *P* ≤ 0.05.

x,y Different superscripts within a row and specific variable indicate a tendency at 0.10 ≥ *P* ≥ 0.05.

1 AST-GOT = aspartate aminotransferase; GGT = γ-glutamyl transferase; ROM = reactive oxygen metabolites; FRAP = ferric-reducing ability of plasma; NEFA = nonesterified fatty acids.

2 Greatest standard error of the mean.

3 Trt = treatment.

**Figure 2:**
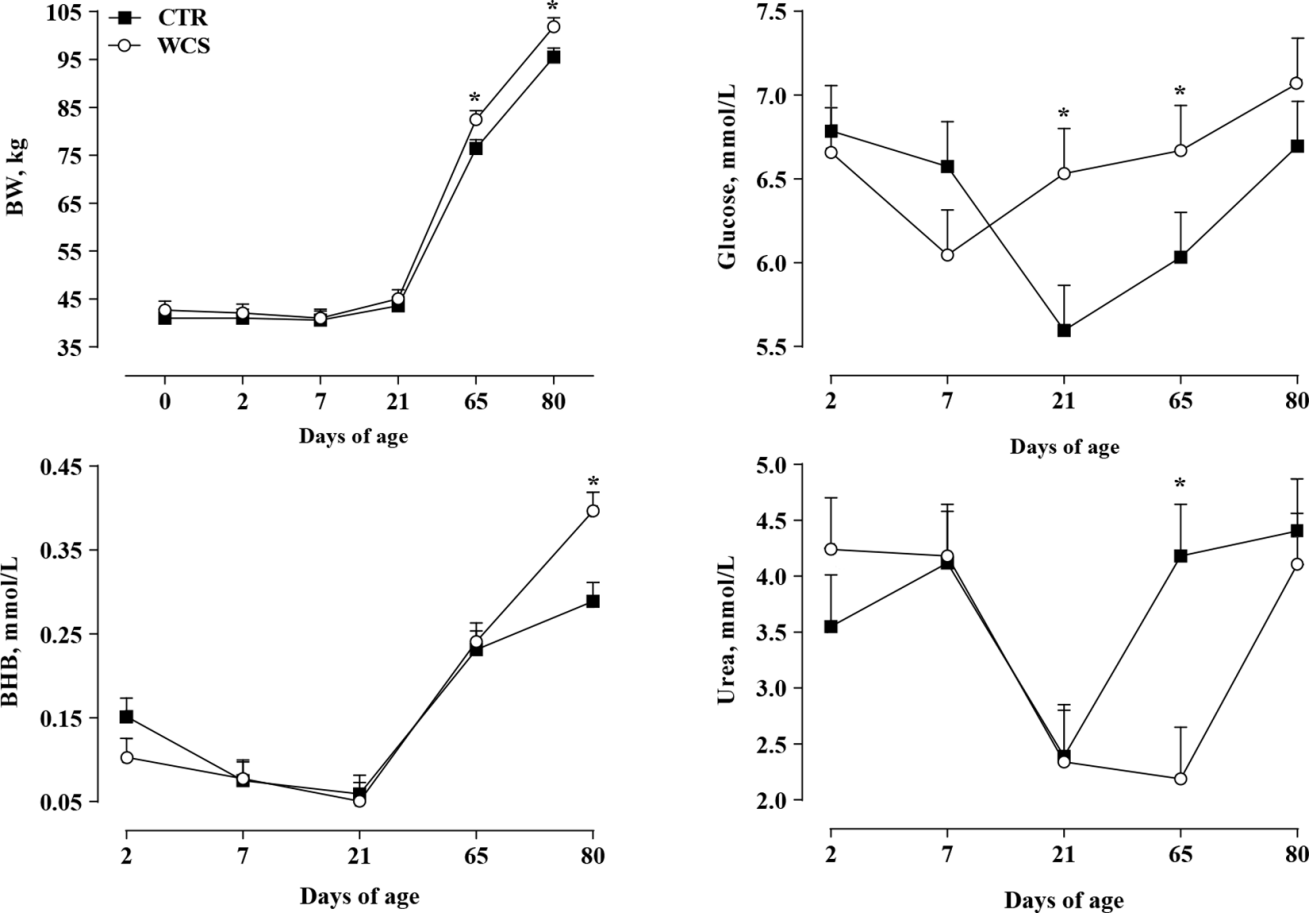
Live BW, glucose, BHB, and urea at 0, 2, 7, 21, 65, and 80 d of calves fed concentrate without (CTR) or with 8% (WCS) of whole cottonseed inclusion. Error bars indicate the SEM. Asterisks indicate days where concentrate intake differed between treatment groups (*P* < 0.05).

Feeding a fiber source in young dairy calves seems beneficial because it leads to rumen development. However, introducing forage during the milk feeding period has long been debated, as the forage is thought to replace concentrate intake and shift rumen fermentation in favor of acetate rather than butyrate production and, thus, delaying rumen papillae development (Tamate et al., 1962; Žitnan et al., 1998). Even though the work of Khan et al. (2011a) reported that chopped hay was able to improve rumen development assessed by blood BHB at 7 and 9 wk of age, the same authors found no differences in BW, although the total DMI of calves fed with chopped hay was markedly greater. This outcome clearly points out that fiber from forage can limit feed efficiency, since forage is less energy dense than calf starter feed. Hence, attempting to find a highly degradable fiber source in young ruminants is still required. Based on this purpose, whole cottonseed, being high in energy, protein, and fiber (Mullenix et al., 2022), may promote growth and stimulate functional development of the rumen especially considering its high-digestible fiber. The results gathered herein confirmed that the inclusion of WCS in the diets of dairy calves led to greater concentrate intake and greater BW after weaning compared with the control group. Anderson et al. (1982) reported positive effects of feeding WCS to young calves. Indeed, calves at 12 wk fed with WCS and WCS paired with hay consumed more feed and were heavier than the control group. Cranston et al. (2006) reported that beef steers fed with whole cottonseed consumed more feed than steers fed with conventional diet, and this result was related to the higher digestible NDF content (+12%) of WCS diet. Thus, we can speculate that the stimulation of rumen walls and forestomach muscle caused by fiber (increase of rumen volume, motility, and muscularization) (Vazquez-Anon et al., 1993; Žitnan et al., 1998) might stimulate the starter intake. In addition, WCS group also showed a better energy balance compared with CTR, likely due to the better degradability and digestibility of WCS, since the WCS is rich in highly digestible fiber, and to the richness in fat and protein. In the present experiment, the greater time spent ruminating in WCS calves immediately after weaning could be related to several causes such as greater intake of solid feeds, greater intake of NDF, which in turn results in better rumen activity. Recently, our group was able to point out the useful approach of measuring rumination time by automatic systems for obtaining quick information on rumen functionality and development (Lopreiato et al., 2020). Taken together, data on performance and rumen activity estimated by the ear-tag showed that the inclusion of WCS can represent a valuable feed ingredient in the calf starter formulation promoting higher concentrate intake, greater BW, and longer rumination time in the immediate postweaning period.

The greater concentration of blood BHB in the WCS group at 80 d was probably due to the increase in starter intake, which also could imply a better rumen development with an increasing activity of microbial population and greater activity of rumen epithelium (Suarez-Mena et al., 2017). If the blood concentration of BHB can be a parameter to easily monitor rumen development (Quigley et al., 1991; Khan et al., 2011b), blood glucose represents, at least in calves, an indicator of the energy status, especially when calves are switched from milk to solid feeding (Baldwin et al., 2004; Khan et al., 2016). Blood glucose is the most affected plasma biomarker during ruminal development. Because of the shift from a glycolytic to a glucogenic liver, blood glucose concentration can be reduced (Lane et al., 2002; Baldwin et al., 2004). According to Haga et al. (2008), when calves increase starter intake, rumen in turn develops and, thus, it results in a decrease in intestinal absorption of dietary glucose. Hence, most of the plasma glucose, especially after weaning, is derived from hepatic gluconeogenesis starting from ruminal propionate (Donkin and Armentano, 1995). Thus, the greater blood glucose concentration in WCS calves could likely reflect the greater concentrate intake and rumen papillae development, which in turn might reflect a possibility of greater VFA absorption (availability of propionate from rumen fermentation).

In addition, the lower blood urea in WCS group, especially at the end of weaning period (65 d), might be associated with a better efficiency of protein utilization at rumen level. We are aware that a complete speculation cannot be pointed out, but considering that WCS calves consumed approximately an average of 0.45 kg/d more than CTR calves in the period 60 to 65 d (resulting also in a greater CP intake), it could be hypothesized that the lower blood urea concentration was due to a greater rumen nitrogen utilization efficiency. Our results confirm that the WCS inclusion in the concentrate can be beneficial during the preweaning period, but mostly in the immediate postweaning, because it increases the overall nitrogen efficiency by rumen. The latter can be provided by an improved fiber digestibility that in turn stimulates the activity of fiber-digesting bacteria. It remains unclear from our data if this outcome is ascribed to a re-modeling of the rumen microbial populations that involves more abundance of fiber and carbohydrate-degrading bacteria and bacteria for butyrate production.

Together with the higher levels of β-carotene and tocopherol and the lower levels of ceruloplasmin and myeloperoxidase, we could assume a lower degree of inflammation and oxidative stress in WCS than CTR group, maybe due to the better rumen function and as a consequence less damage at the level of the epithelia, which involves less risk of compromising gut permeability. In fact, myeloperoxidase is an important enzyme involved in the production of ROS during inflammatory processes (Kirschvink and Lekeux, 2002) and ceruloplasmin is an acute-phase protein whose increase is associated with inflammation or infection (Hajimohammadi et al., 2013). Regarding liver function biomarkers, paraoxonase levels resulted greater in WCS group than CTR. Paraoxonase is a negative phase acute protein (Feingold et al., 1998; Bionaz et al., 2007), which is reduced by liver along with albumin and lipoprotein during acute-phase response (Bionaz et al., 2007; Bertoni et al., 2008). Furthermore, the increase of PON level in plasma is associated with increasing values of total cholesterol (Bionaz et al., 2007; Ferronato et al., 2022). Thus, the higher levels of PON and tocopherol support a general health status due to a better liver metabolism and oxidative balance of the group supplemented with WCS.

Possible limitations to the present study include the low sample size and the use of straw as bedding. Considering our results, no treatment effect was noted before the beginning of the weaning period. Differences between groups were mostly during and after weaning, when hay and TMR were provided ad libitum. Therefore, intake of straw was unlikely to determine major effects on the outcomes measured. Anyway, results of this study should be interpreted carefully and intended as preliminary.

In conclusion, feeding strategies and good nutritional programs have a significant impact for the future of the dairy industry. In the present study, including WCS in the concentrate had positive effects on growth performance through increasing starter intake, BW, rumen development (as indicated by blood BHB), and rumination. Moreover, the greater levels of BHB, glucose, fructosamine, PON, tocopherol, and β-carotene could reflect a positive metabolic response, together with a low stress response supported by lower blood levels of ceruloplasmin and myeloperoxidase, evidencing a potential better health status. Hence, including WCS in calf starter during preweaning and weaning ensures a good feeding management strategy of calves' system, especially in the transition from milk to only solid feeds, which is beneficial for promoting development of the gut and rumination behavior in young calves.
